# Visualizing inconsistency in network meta-analysis by independent path decomposition

**DOI:** 10.1186/1471-2288-14-131

**Published:** 2014-12-16

**Authors:** Ulrike Krahn, Harald Binder, Jochem König

**Affiliations:** Institute of Medical Informatics, Biometry and Epidemiology, University Hospital, University Duisburg-Essen, Hufelandstr. 55, 45122 Essen, Germany; Institute of Medical Biostatistics, Epidemiology and Informatics, University Medical Center Johannes Gutenberg University Mainz, Langenbeckstr. 1, 55101 Mainz, Germany

**Keywords:** Network meta-analysis, Multiple treatments comparison meta-analysis, Mixed treatment comparison meta-analysis, Inconsistency, Influence diagnostics, Forest plot

## Abstract

**Background:**

In network meta-analysis, several alternative treatments can be compared by pooling the evidence of all randomised comparisons made in different studies. Incorporated indirect conclusions require a consistent network of treatment effects. An assessment of this assumption and of the influence of deviations is fundamental for the validity evaluation.

**Methods:**

We show that network estimates for single pairwise treatment comparisons can be approximated by the evidence of a subnet that is decomposable into independent paths. Path-based estimates and the estimate of the residual evidence can be used with their contribution to the network estimate to set up a forest plot for the consistency assessment. Using a network meta-analysis of twelve antidepressants and controlled perturbations in the real and constructed consistent data, we discuss the consistency assessment by the independent path decomposition in contrast to an approach using a recently presented graphical tool, the net heat plot. In addition, we define influence functions that describe how changes in study effects are translated into network estimates.

**Results:**

While the consistency assessment by the net heat plot comprises all network estimates, an independent path decomposition and visualisation in a forest plot is tailored to one specific treatment comparison. It allows for the recognition as to whether inconsistencies between different paths of evidence and outlier effects do affect the considered treatment comparison.

**Conclusions:**

The approximation of the network estimate for a single comparison by the evidence of a subnet and the visualisation of the decomposition into independent paths provide the applicability of a graphical validation instrument that is known from classical meta-analysis.

## Background

In medical practice, several treatments are frequently suitable for a single indication, but often only two or three of them are directly compared in one study. Network meta-analysis is an approach that combines the information from all clinical trials on any of the treatments based on the assumption of consistent treatment effects and the inclusion of indirect comparisons (for an overview see e.g. [1]). The evaluation of the consistency and the influence of possible deviations of this assumption on network estimates plays an important role in the validation of results [2], especially as more complex models are fitted [3]. In classical meta-analysis, a forest plot of study effects and the pooled estimate offers the visualisation of outlier effects and their contribution to the aggregated treatment effect [4]. In network meta-analysis, the homogeneity between studies of each pairwise treatment comparison can also be analysed using forest plots. However, to assess the consistency assumption in the network, study-based forest plots are not directly applicable, since for different pairwise treatment comparisons various effects are expected [1]. Therefore, a generalised forest plot approach is needed.

Salanti et al. [5] graphically analysed consistency using a forest plot of the differences between direct and indirect evidence in single network loops. In a forest plot, estimates based on direct, indirect (obtained by back-calculation or node-splitting [6]), and combined evidence for one treatment comparison can be compared [1, 2] without reflecting detailed sources of potential inconsistencies. Tools for regression diagnostics like the plot of posterior mean deviance of individual data points [6, 7], the plot of leverage against the residual deviance for each data point [8], or the assessment of PRESS residuals and studentised residuals [9] allow for the singling out of individual studies or a set of studies that compared the same treatments whose direct evidence is badly fitted and may be responsible for heterogeneity or inconsistency. For an influence analysis of potentially inconsistent direct evidence, the contribution of direct evidence to network estimates has to be taken into account. Krahn et al. [10] proposed a matrix visualisation, called net heat plot, that highlights hot spots of inconsistency between specific direct evidence in the whole network and renders possible drivers transparent.

None of these approaches can offer all the analytical capacities available in a classical meta-analysis forest plot to a consistency assessment in a network meta-analysis: namely the composition of the network effect estimate based on direct evidence, the consistency between different evidence sources, outlier observations and the influence on aggregated treatment effect estimates.

In the following, we show that a single network effect estimate (e.g. for the comparison between treatments *A* and *B*) can also be approximated by the evidence of a subnet that is decomposable into independent paths and can be visualised in a forest plot. For a consistency investigation, it allows the visualisation of the contribution of each independent path as well as that of the residual evidence in combination with their corresponding treatment effect estimate. Due to the additional display of the network-based treatment effect, an influence assessment of deviating direct evidence is possible. We discuss this tool for consistency and influence assessment in contrast to the net heat plot [10] by using an evidence network of twelve antidepressants. We assess controlled perturbations in a constructed, consistent dataset of the example and subsequently in the real data.

This article is structured as follows: We start with the data example. In the Methods section, we discuss the influence of direct evidence in network meta-analysis and derive an influence function as well as the concept of a decomposable subnet approximation and its visualisation. In the Results section, we apply our approach to the data and compare it with net heat plot results. The paper concludes with a discussion of our findings.

## Application

**Figure 1:**
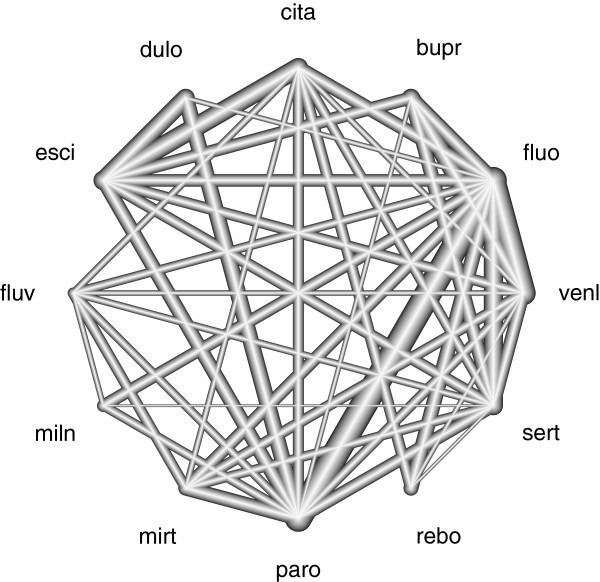
**Evidence network of the antidepressants example.** The lines display the observed treatment comparisons. The thickness of a line is proportional to the inverse standard error of the directly estimated treatment effect, which is aggregated over all studies including the two respective treatments.

As a data example, we consider a network meta-analysis performed by Cipriani et al. [11] (for data availability see [11]). In this analysis, twelve antidepressants (see Table 1) are examined regarding response, defined as a reduction of at least 50% from the baseline of the depression rating score after eight weeks. The odds ratio (OR) was used as effect measure. In total, 111 randomised trials are included in this network meta-analysis comprising 109 two-armed trials and two three-armed trials. Figure 1 shows the complexity of the network; the assessment of inconsistency and influence of perturbations pose a challenge.

**Table 1:** **Twelve antidepressants examined in the network meta-analysis published by Cipriani et al.** [11]

Treatment	Abbreviation	Treatment	Abbreviation
Bupropion	bupr	Milnacipran	miln
Citalopram	cita	Mirtazapine	mirt
Duloxetine	dulo	Paroxetine	paro
Escitalopram	esci	Reboxetine	rebo
Fluoxetine	fluo	Sertraline	sert
Fluvoxamine	fluv	Venlafaxine	venl

## Methods

In the following, we briefly present a fixed effects model for network meta-analysis that has already been explained in more detail by Krahn et al. [10] and in this context analyse how changes in study effects are translated into network estimates. We introduce a simplification of meta-analysis networks into decomposable subnets that allows the application of a forest plot for a single network-based treatment effect.

### Fixed effects model in network meta-analysis

We consider a network meta-analysis for *T*+1 treatments *A*_0_,…,*A*_*T*_ that are compared in a set of studies building a connected evidence network as for instance shown in Figure 1. Assuming consistency, all pairwise treatment effects in the network are uniquely determined by the *T* basic contrasts to a reference treatment *A*_0_, which we denote by the vector *θ*^net^. Characterising each study by the investigated set of treatments (called design in the context of network meta-analysis [12, 13]), it has been demonstrated that the generalised least squares estimation for a fixed effect model can be partitioned into two steps [10]: Firstly, the evidence is pooled for each design *d* (*d*=1,…,*D*) to get an aggregated treatment effect  (e.g. an aggregated log(OR)) with covariance . (In the case that *d* is a design of one or more *N*_*d*_-armed studies,  is a vector of length (*N*_*d*_−1) and  a matrix of size (*N*_*d*_−1)×(*N*_*d*_−1); for a detailed explanation see [10]). Secondly, the vector of all direct treatment effect estimates  and the known covariance matrix  are used to fit the model:
1

The design matrix *X* comprises one column for each treatment *A*_1_,…,*A*_*T*_ and one row for each design and each comparison with a design-specific reference (i.e.  rows). If included in a design, this design-specific reference equals *A*_0_. The entries of *X* have value 1 in the column corresponding to the treatment compared with the reference, -1 in the column corresponding to a design-specific reference whenever it is not *A*_0_, and 0 else. The vector of all error terms *ε* is assumed to be independent across designs and normally distributed with covariance matrix *V*.

The vector of basic contrasts *θ*^net^ can then be estimated by generalised least squares as


with predicted effects . Thereby, the matrix *H*=*XB* projects the estimates based on direct evidence to the network estimates and provides in each row the linear coefficients for one network estimate. Thus, a network estimate of treatment *u* versus treatment *t* is composed as follows:


where *H*_*tu*,._ denotes one row vector, and *h*_*tu*,*d*_ denotes one entry (or in the case that *d* is a design of one or more *N*_*d*_-armed studies, a transposed partial row vector of length (*N*_*d*_−1)) of *H*.

### Influence of single studies or designs in network meta-analysis

In classical meta-analysis, a study with a highly deviating treatment effect estimate may have a strong influence on the aggregated effect estimate depending on its weighting in the estimation process. Using generalised least squares estimation, the weight is proportional to the inverse variance of the treatment effect estimate.

In network meta-analysis, the weighting of a study in one network estimate  depends not only on its corresponding precision, but also on the network structure: Study *s* of design *d* with covariance matrix *V*_*s*_ contributes in model (1) to the aggregated treatment effect estimate  with weight , where  denotes the set of all studies with design *d*. The direct treatment effect estimate  in turn drives a network estimate  by the value of the corresponding element *h*_*tu*,*d*_ of matrix *H*. So, the contribution of study *s* is given by


If treatment effect estimate  of design *d* is inconsistent with the treatment effect estimates of the remaining network, the question arises as to how this influences a network estimate . Therefore, we define an influence function as the change in a network estimate when the direct treatment effect estimate  is shifted by *δ*. For a design of two-armed studies, this means if  where *e*_*d*_ denotes the unit vector with 1 at position *d* and 0 elsewhere,


In the case of multi-armed designs, influence functions for each pairwise comparison can be defined similarly.

### Net heat plot

To assess consistency and influence in a classical meta-analysis, study weights and deviations between study effects and the aggregated treatment effect are, for example, visualised in a forest plot. For a similar analysis in network meta-analysis, a so-called net heat plot has been proposed [10]. In a matrix visualisation (see examples in the Results section), the contribution of the aggregated direct evidence of each design (in a column) to each network estimate (in a row) is shown by the area of gray squares. The greater the area of a square, the greater the contribution of the respective direct evidence to the network estimate in the row. In combination with this evidence flow, a heat matrix for assessing the inconsistency in the network (quantified by a generalized Cochran’s *Q* statistic) is shown: The colours on the diagonal represent the inconsistency contribution, the summand of the Cochran’s *Q* statistic, of the corresponding design. The colours on the off-diagonal are associated with the change in inconsistency between direct and indirect evidence in a network estimate in the row after relaxing the consistency assumption for the effect of one design in the column. Blue colours indicate an increase and warm colours a decrease. In the case that the colour vector of a column is equal to the colour vector on the diagonal, the detachment of the respective design resolves the inconsistency in the whole network.

The detaching of single designs is similar to the node splitting technique of Dias et al. [8], but the net heat plot approach additionally tracks the influence of each design on the fit on all other designs and visualises the successive detaching of each designs in one plot. In contrast, the approach to the consistency assessment presented in the following allows for the application of a forest plot for one specific treatment comparison.

### Decomposable subnets

For the assessment of consistency and influence in one network-based comparison, we use a simplification of the network into a decomposable subnet. Firstly, we will describe our approach for networks formed only by two-armed studies and we will refer to the case of multi-armed studies in Section ‘Multi-armed designs’.

A treatment effect estimate between treatments *t* and *u* is most easily assessable if the underlying network is entirely composed of independent paths between the treatment nodes *u* and *t* (see also [14]). Two paths are defined as independent if they do not share any edges. Each path-based estimate can be formulated by the sum over the intermediate effect estimates. For example, the indirect estimate for treatment effect *u* versus *t* via treatment *v* can be calculated by  [15]. Path based estimates of independent paths are uncorrelated because they are based on independent evidence. The variance is obtained by summing up the direct estimate variances as described by Bucher et al. [15]. All path-based estimates are combined as a weighted sum with weights proportional to their inverse variances. For assessing the consistency, we display this aggregation step in a forest plot where each row represents a path-based effect estimate and each path is identified by its unique list of intermediate treatments.

If the given network is not decomposable as described above, we consider the set of all decomposable subnetworks and choose one with the most precise resulting effect estimate. Although challenging in general, it is often easy to find a well approximating decomposable subnet just by selecting all paths containing no or only one intermediate treatment. Note that in a network represented by a complete graph with direct effect estimates of constant precision, the best approximating decomposable subnet (i.e. with the most precise resulting effect estimate) is uniquely defined by the direct path and all two-step indirect paths [16]. The resulting estimate is identical to the network-based estimate.

Once an approximating decomposition is achieved, we denote the corresponding network estimate as . The residual evidence of the network can be defined as the pseudo effect estimate


with precision


(see analogously the back-calculation in [8]). Then, the network-based estimate is the weighted sum


with


This weight describes the proportion of evidence for the given effect that is contained in the subnetwork. In the following, we refer to it as approximating evidence proportion. Note that the variance of the difference between the network and the subnetwork-based estimate is


which can be used to straightforwardly define a *Z* statistic to test for consistency between the approximation and the network-based estimate (by analogy with e.g. [16]).

#### Forest plot

We use the path-based effect estimates and the pseudo estimate together with their precisions to hierarchically set up a forest plot that captures how the subnetwork is composed of independent path-based estimates and, at a higher level, how the subnet contributes to the whole network-based estimate (see examples in the Results section). The resulting overall Cochran‘s *Q* statistic has degrees of freedom (df) equal to the number of independent paths. It is similar but not identical to the inconsistency *Q* [10]. It captures solely that aspects of inconsistency that have consequences for the uncertainty about the effect estimate  The weights in the forest plot can be interpreted as the proportion at which a perturbation in any direct estimate contributing to that path is translated into a bias of the network estimate  If the weight of the residual evidence is small, no perturbation outside the subnet at any plausible scale is able to substantially affect the network estimate.

#### Iterated shortest path algorithm

The enumeration of all decomposable subnets may be too cumbersome. We therefore propose a simple algorithm that aims to find a good, but not always the best, approximating decomposable subnetwork. We therefore define the distance between two adjacent nodes as the variance of the corresponding direct effect estimate, and the length of a path is identical to the variance of the path-based effect estimate. The proposed algorithm is as follows:

1. Start selecting the shortest path between nodes *t* and *u* and eliminate all its edges [17].
2. Iterate until the nodes *t* and *u* are no longer connected.

The set of all eventually selected paths make up the approximating sub-network and its independent path decomposition.

#### Multi-armed designs

Different strategies can be used for multi-armed designs: Firstly, they can be kept out of all paths of a subnetwork of independent paths. Secondly, one convenient treatment comparison for each multi-armed design, for example, the comparison between A and B for the multi-armed design ABC, can be allowed to potentially contribute in combination with all two-armed studies of design AB. This implicitly assumes that direct evidence for the relative effect *θ*_*tu*_ does not depend on the remaining treatments investigated in the same study. A further strategy is to use at most one comparison (e.g. AB) of each multi-armed design separated from all two-armed studies (of design AB) and to build extra paths with these comparisons if possible. For the examples discussed in the following, we use the first strategy.

### Construction of validation datasets

**Figure 2:**
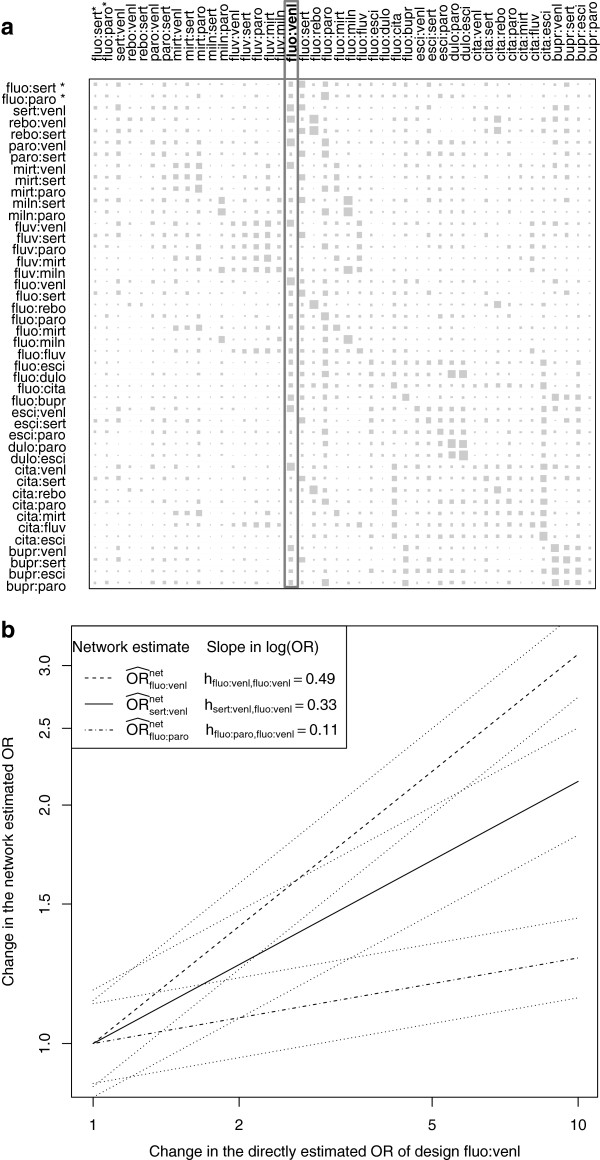
**Influence analysis in the antidepressants example. a)** Visualised *H* matrix: The contribution of the direct estimate of one design in the column to a network estimate in the row is shown by the area of the corresponding gray square. The two contrasts of the two three-armed studies with design fluo:paro:sert are marked by ^∗^. A design whose direct evidence contributes greatly to the network estimate of some other designs is framed. **b)** Three exemplary influence functions: The influence of direct evidence of design fluo:venl on the network estimates with designs fluo:venl (dashed line), sert:venl (solid line), and fluo:paro (dashed and dotted line) and their corresponding 95% confidence intervals (dotted lines) is shown.

In the Results section, we illustrate the influence analysis and the decomposition of an evidence network into independent paths based on the antidepressants example. For validating the analysis capabilities for the proposed path-based forest plot assessment in contrast to the net-heat plot approach, we use controlled perturbations, firstly in a constructed, consistent dataset and then in the real data. We constructed the consistent network of treatment effects based on the antidepressants example by setting the OR of all studies to one and retaining all standard errors. The corresponding net heat matrix is drawn in Figure 2a. Perturbations were effected by adding a *δ*= log(2) to selected log odds ratios .

## Results

We demonstrate the influence analysis as well as the approach of the independent path decomposition and its visualisation by applying the methods to the antidepressants example. The analysis capability by the independent path decomposition is discussed in comparison to the net heat plot approach using controlled perturbations in the consistent validation dataset as well as in the real data. To produce the forest plots and the net heat plots, we used functions of the R packages meta [18] and netmeta [19]. Software instructions and an R function for the iterated shortest path algorithm are available on the website http://www.unimedizin-mainz.de/imbei/biometrie/software.html.

### Influence analysis

The contribution of all design-specific direct evidence to the network estimates in the antidepressants example is visualised in Figure 2a. The areas of the gray squares are determined by the elements of the projection matrix *H* of the underlying model (as well as in [10]). As seen from the column of design fluo:venl for example, the corresponding direct estimate represents a large source of evidence as it drives the network estimates for many treatment contrasts. In particular, (as visualised by the area of the gray square in the diagonal element fluo:venl) the network estimate for fluo:venl is based on 49% direct evidence of 11 studies with inverse variance weights between 2.1% and 16.3%. The contribution of single studies to the network estimate is therefore beween 1% and 8%.

Figure 2b shows the influence of the direct evidence of design fluo:venl on three exemplarily chosen network estimates that could be important if the evidence of design fluo:venl deviates from the assumption of consistency. Here, the relative change in the network estimate against the change in the direct estimate is plotted. The functions are linear on a log scale with slope determined by the *H* matrix entries. For instance, doubling the OR of the direct estimate with design fluo:venl (, with *δ*= log(2)) changes the three network estimates shown by 8%, 26%, and 40%. In the constructed, consistent data example (see Section ‘Construction of validation datasets’), this means that a directly estimated OR in design fluo:venl of 2.00 [1.67;2.39] instead of 1.00 [0.84;1.2] shifts the network estimates for comparisons fluo:paro, sert:venl, fluo:venl from 1.00 [0.89;1.12], 1.00 [0.86;1.17], and 1.00 [0.88;1.13] to 1.08 [0.96;1.21], 1.26 [1.08;1.47], and 1.41 [1.24;1.59]. Such a deviation of the direct evidence of design fluo:venl from the assumption of consistency would bias the network estimates of comparisons sert:venl and fluo:venl to at 5% level significant treatment effects.

### Approximation by independent path decomposition

The network of antidepressants shown in Figure 1 is highly complex and interconnected. It has 42 edges supported by direct evidence including two three-armed studies. But a lot of treatment comparison estimates in this network meta-analysis are dominated by direct and simple indirect evidence including only one intermediate treatment.

**Figure 3:**
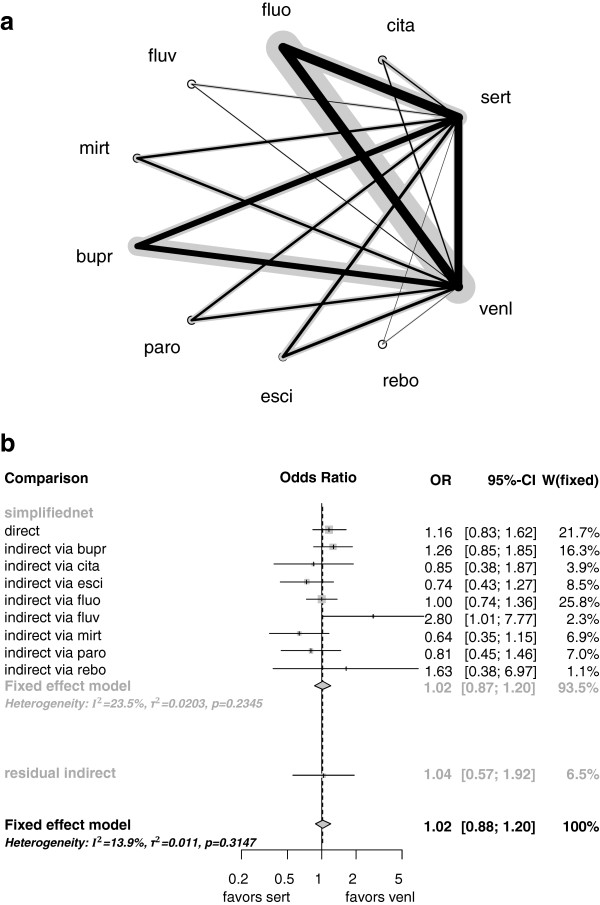
**Approximation by independent path decomposition for network estimate of comparison sert:venl in the antidepressants example. a)** Approximating decomposable subnet. The thickness of a gray line is proportional to the inverse standard error of the corresponding directly estimated treatment effect. The thickness of a black line represents the contribution of direct evidence to network estimate sert:venl. **b)** Forest plot of the independent path subnet and the residual evidence.

For example, the network estimate of comparison sert:venl is primarily based on direct evidence with 22% and on eight indirect comparisons including only one intermediate treatment (via bupr, cita, esci, fluo, fluv, mirt, paro, and rebo). This independent path decomposition is shown in Figure 3a and is the result of the iterative shortest path algorithm. The corresponding forest plot is displayed in Figure 3b. The approximation by the independent paths provides 93.5% of the whole network’s evidence regarding the estimation of the treatment comparison sert:venl.

This display shows fairly consistent independent path-based estimates with exception of the indirectly estimated treatment effect via fluv with OR 2.8 [1.01;7.77]. Since the contribution of this estimate is 2.3%, the network estimate is only influenced a little. The pseudo estimate of the residual evidence is in accordance with the approximation, and the *Q* statistic of 10.46 (df=9, p=0.31) between the different sources of evidence indicates only slight inconsistency.

We generated approximations for all 66 pairwise treatment comparisons in the network. The median approximating evidence proportion was 85% (with range of 55-97%).

### Validation of the sensitivity to perturbations in contrast to the net heat plot approach

To compare the consistency analysis in forest plots of independent paths in contrast to the net heat plot approach, we perturbed chosen direct treatment effects in the constructed, consistent dataset as described above.

**Figure 4:**
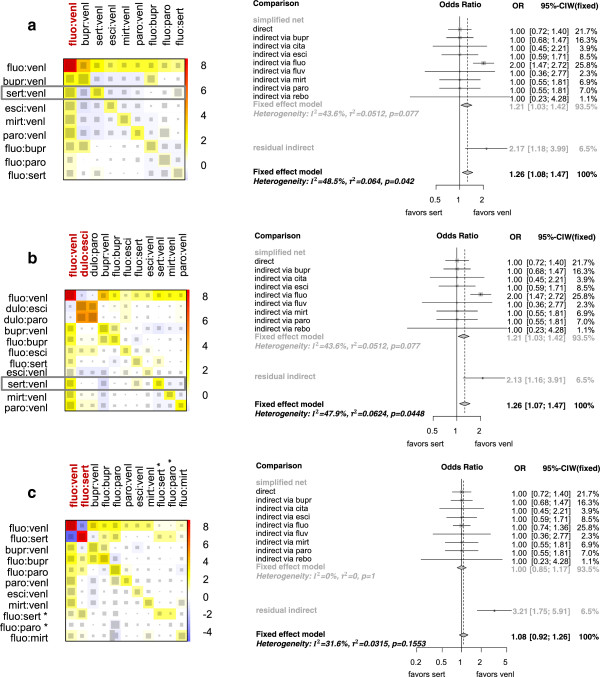
**Approximation by independent path decomposition versus net heat plot approach in the constructed examples after perturbing the direct estimates in a) of fluo:venl, in b) of fluo:venl and dulo:esci, and in c) of fluo:venl and fluo:sert (column red marked respectively) by inflating the corresponding OR by a factor of two.** Left: Net heat plots in which the area of a gray square displays the contribution of the direct estimate of one design in the column to a network estimate in the row. The colours on the diagonal represent the inconsistency contribution of the corresponding design. The colours on the off-diagonal are associated with the change in inconsistency between direct and indirect evidence in a network estimate in the row after relaxing the consistency assumption for the effect of one design in the column. Blue colours indicate an increase and warm colours a decrease. Only rows and columns of the net heat plots are shown, where the maximal absolute entry exceeds or is equal one. The two contrasts of the three-armed studies are marked by ^∗^. Right: Forest plots for the network estimate of comparison sert:venl based on the independent path decomposition of Figure 3a in the three constructed examples.

At first, we perturbed the direct treatment effect estimate of design fluo:venl by inflating the OR by a factor of two. In the net heat plot, this results in an associated red-coloured diagonal element that depicts the contribution of design fluo:venl to the inconsistency in the network (see Figure 4a). Other designs, for example sert:venl, contribute to the inconsistency statistic as well, albeit in attenuated form, which can be seen by the corresponding yellow-coloured diagonal elements. This is because their network estimates are largely driven by the direct treatment effect of design fluo:venl (see the gray squares in column fluo:venl), and these network estimates are also affected. Inspecting the warm-coloured off-diagonal elements, inconsistency between the direct evidence of designs fluo:venl and bupr:venl as well between fluo:venl and fluo:bupr can be observed. Since only the elements in the column of fluo:venl are coloured the same as the corresponding diagonal elements, a complete elimination of inconsistency in the whole network is only reached after relaxing the consistency assumption for design fluo:venl.

On the right side of Figure 4a, the forest plot for the network estimate of comparison sert:venl based on the independent path decomposition of Figure 3a is shown. Since we constructed the dataset by setting the OR of all studies to one (with exception of the OR of studies with design fluo:venl in this perturbed case), the network estimate for comparison sert:venl, shown by the diamond on the bottom of the forest plot, should be equal to one. But it can be seen that the indirect estimate via fluo (OR: ) as well as the pseudo effect estimate of the residual evidence is affected by the perturbed direct treatment effect estimate of design fluo:venl. This, in turn, influences the network estimate of comparison sert:venl, since the indirect estimate via fluo comprises 26% of the estimate.

In perturbation setting two, when we perturbed not only the direct treatment effect of design fluo:venl, but also of design dulo:esci to the same extent, we can observe the largest inconsistency contributions in the net heat plot in Figure 4b for these designs as well as for design dulo:paro. The blue-coloured elements in the upper left corner indicate that the direct effect of design fluo:venl and that of dulo:esci (or alternatively dulo:paro) support each other. This means the detachment of one of both designs from the network estimation increases the residual of the other design and the inconsistency in the network can be eliminated in neither of the detachments. Since the adjacent edges corresponding to designs dulo:esci and dulo:paro are part of an essentially non-branching path, the residuals resulting from the detachment of one of both designs are highly correlated, and the two corresponding columns contains very similarly coloured elements. Because the direct evidence of design dulo:esci drives only a little of the network estimate of comparison sert:venl, which can be seen by the little gray square in column dulo:esci and row sert:venl, the additional perturbation of the effect of design dulo:esci hardly changes the network estimate of comparison sert:venl.

This is duly reflected in the accompanying forest plot. Due to the small contribution of the direct evidence of design dulo:esci to the network estimate of comparison sert:venl, the edge corresponding to design dulo:esci is not part of the subnet that approximates the network estimate of comparison sert:venl, and the perturbation is only slightly recognisable in the forest plot by the small change of the residual evidence.

In perturbation setting three, in addition to the direct treatment effect of design fluo:venl, we perturbed the effect of design fluo:sert by inflating the OR by a factor of two. An indirect effect estimate of comparison sert:venl via fluo therefore also results in an OR of 1 as in the unperturbed case (). The net heat plot in Figure 4c indicates that both perturbations take effect in the same direction (the direct effects of both designs support each other, as shown by the blue-coloured elements), whereas in the forest plot only a small change in the pseudo effect estimate of the residual evidence is recognisable, and the indirect estimate via fluo is not affected at all. Thus, the forest plot clearly reveals that both perturbations taken together are not relevant for the comparison sert:venl.

In summary, in the forest plot for one network estimate, an inconsistent direct estimate is only identified if its edge is part of the approximating subnet and is not dissolved within an independent path. That is, the forest plot is selectively sensitive to biases that affect the considered treatment comparison. In contrast the net heat plot summarises the network drivers and inconsistencies in the whole network.

**Figure 5:**
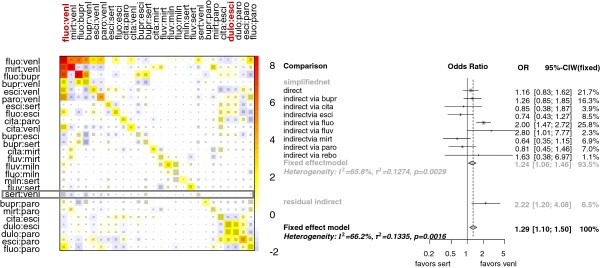
**Approximation by independent path decomposition versus net heat plot approach in the antidepressants example after perturbing the red-marked direct estimates in the columns.** Left: Net heat plot in which only rows and columns are shown, where the maximal absolute entry exceeds or is equal one. Right: Forest plot for the network estimate of comparison sert:venl.

Using the real data of the antidepressants network-meta analysis after perturbing the directly estimated odds ratios for designs fluo:venl and dulo:esci by a factor of two, we can also see that both perturbations are detected by the net heat plot in Figure 5. But as seen in the forest plot for comparison sert:venl, the first one almost exclusively affects the network estimate and the consistency between different evidence sources.

## Discussion and conclusions

In network meta-analysis, evidence from different designs contributes to a comparison between two treatments. For the consistency assessment between the effects of different evidence sources, we have provided a visualisation of an approximating independent path decomposition by forest plots and have investigated its performance in comparison to that of the net heat plot. We have shown for the example of the highly interconnected network of twelve antidepressants that most network-based treatment comparisons are approximated well by the evidence of independent paths. The proposed forest plot discloses and summarises the essentials of a given network-based treatment comparison: the weight given to each path, the consistency between all paths, the comparison between the estimate based on the approximation and on the whole network, and the residual evidence that is not included in the approximation is condensed into a pseudo estimate. The heterogeneity reported in this forest plot captures these aspects of the inconsistency, which are crucial for the considered comparison.

By perturbing the antidepressants network, after making it artificially perfectly consistent, we have shown how different kinds of perturbation show up as drivers of inconsistency in the net heat plot or as outlying path-specific evidence in the forest plot. While the net heat plot is sensitive to all kinds of perturbation that sufficiently inflate Cochran’s Q statistic for inconsistency, the forest plot indeed revealed to be selectively sensitive to perturbations that are influential to the considered comparison.

Independent path approximations should be considered only if they capture, say, more than 80% of the network evidence. If this is not the case, the complete network should be inspected. The flow of evidence can be displayed as a graph as outlined in [16], and influential designs can be sought for in the net heat plot. A forest plot that exhibits consistent and balanced evidence from several independent paths and that is additionally in accordance with the residual evidence may indeed be more convincing than a simple meta-analysis of direct comparisons (as similarly argued in [20]).

Paths independence can be defined in two ways, edge independence and vertex independence. In both definitions, the path-based effect estimates are uncorrelated. We have applied edge independence. Note that in an edge independent path decomposition, the estimate based on the subnet may be different from the estimate based on the independent paths if some paths share a common intermediate vertex. Two paths may contain some common intermediate vertices, and the forest plot implicitly splits these nodes. As a consequence, the subnet has more inconsistency degrees of freedom [10] than the corresponding forest plot. For the antidepressants network, on average only 2% of precision is lost by the approximation based on independent paths instead of the subnet.

We focused on the fixed effects model in our exposition, because inconsistency is most easily detectable then [1], but note that the proposed forest plot can be adapted to random effects models by assuming that the heterogeneity variance is known and fixed and by adding appropriate elements to the covariance matrix of direct effect estimates. The heterogeneity variance parameters would have to be assessed using the study level data (see e.g. [21] for various options to model the heterogeneity covariance structure). Then a forest plot is set up for the resulting independent paths formally using the fixed effects option but based on increased variances of the direct effect estimates. Random effects parameters can be estimated e.g. via the method of moments, by restricted maximum likelihood or by Bayesian methods. If the resulting point estimates of heterogeneity variance parameters are plugged in, our diagnostic tools are valuable for all these approaches.

In the literature, various methods have been discussed for the assessment of outliers and influence. In principle, all methods introduced for linear regression [22, 23] can be used [2, 9]. For network meta-analysis, plots of deviance residuals [24] have been discussed [8] as well plots of squared Pearson residuals [25]. At an aggregate level, regression diagnostics amount to analysis of inconsistency, with index plots of leverages and residuals [26], concepts like node-splitting [8], and design-by-treatment interaction [12, 13]. None of these approaches focuses on the meaning of inconsistency-generating evidence for a specific treatment comparison and none offers all the analytical capacities known from the forest plot in classical meta-analysis for visualising the composition of evidence and identifying potential discrepancies.

Our proposed methods are confined to comparisons that are well-approximated by an estimate based on an independent path decomposition. In complex networks, comparisons that do not fit into this scheme may well be assessed by defining an approximating subnet that is more complex than a decomposable subnet, but much less complex than the whole network. In the antidepressants example more than half of all edges have weights less than 1/44, which is one fourth of the weights seen in a complete and balanced network of 12 treatments. Only a few percentages loss in precision should result from omitting these edges. Resorting to a subnet estimate if it captures more than 95% of the evidence for one comparison could be a remedy to the model uncertainty, e.g. with regard to the network size that was discussed in [27].

In conclusion, we have introduced forest plots of independent path decompositions for the assessment of consistency in complex meta-analytic networks as well. We have seen that this graphical presentation known from classical meta-analysis captures the essentials of a network-based treatment comparison and discloses both the composition of evidence and sources of potential inconsistency relevant for the considered comparison.
